# The Hidden Anatomy of a Lower Premolar With Two Canals

**DOI:** 10.7759/cureus.27393

**Published:** 2022-07-28

**Authors:** Akash Sibal, Anuja Ikhar, Shriya R Singi, Mayur B Wanjari

**Affiliations:** 1 Department of Conservative Dentistry and Endodontics, Sharad Pawar Dental College, Datta Meghe Institute of Medical Sciences, Wardha, IND; 2 Epidemiology and Public Health, Jawaharlal Nehru Medical College, Datta Meghe Institute of Medical Sciences, Wardha, IND

**Keywords:** endodontics, number of roots, number of canals, mandibular second premolar, root canal morphology

## Abstract

Variations in the root canal configuration are a great challenge for the endodontist during endodontic procedures. This necessitates the understanding of canal morphology before initiating the treatment. Mandibular second premolars have been always studied to have only a single canal in their root. The present case reported shows an unusual, rare occurrence of an extra canal in the single-rooted second premolar. The patient reported pain associated with #35 which was due to deep distant proximal caries. The second canal was revealed during the intraoperative phase and was successfully treated. The current case adds an important insight to the existing literature related to diverse canal configurations in lower premolars.

## Introduction

Residual microorganisms or debris in the canals that are improperly cleaned or neglected are one of the most common causes of endodontic treatment failures. The purpose of the root canal treatment is complete biomechanical preparation of the entire canal and consecutive obturation using an inert material [1]. However, a clinician may experience certain deviations from the standard root canal configuration. Therefore, a comprehensive understanding of the root and canal system is a prime requisite for the success of endodontic procedures. Mandibular premolars have been considered to impose significant challenges during root canal treatment due to the vulnerable root canal patterns [2]. Mandibular second premolars are universally well-known for possessing a single root with a single canal. Additional canals may be discerned accidentally during treatment procedures which can be linked with complications in the endodontics treatment [3]. To avoid such circumstances, dental clinicians should be aware of such significant variations in the canal configuration of the root canal system. The occurrence of two canals in the second premolar has also been reported, but the frequency was approximately just 2.8% which is very rare or not clinically significant [4]. The present case highlights an unusual encounter of an extra canal in the single-rooted mandibular second premolar.

## Case presentation

**Figure 1:**
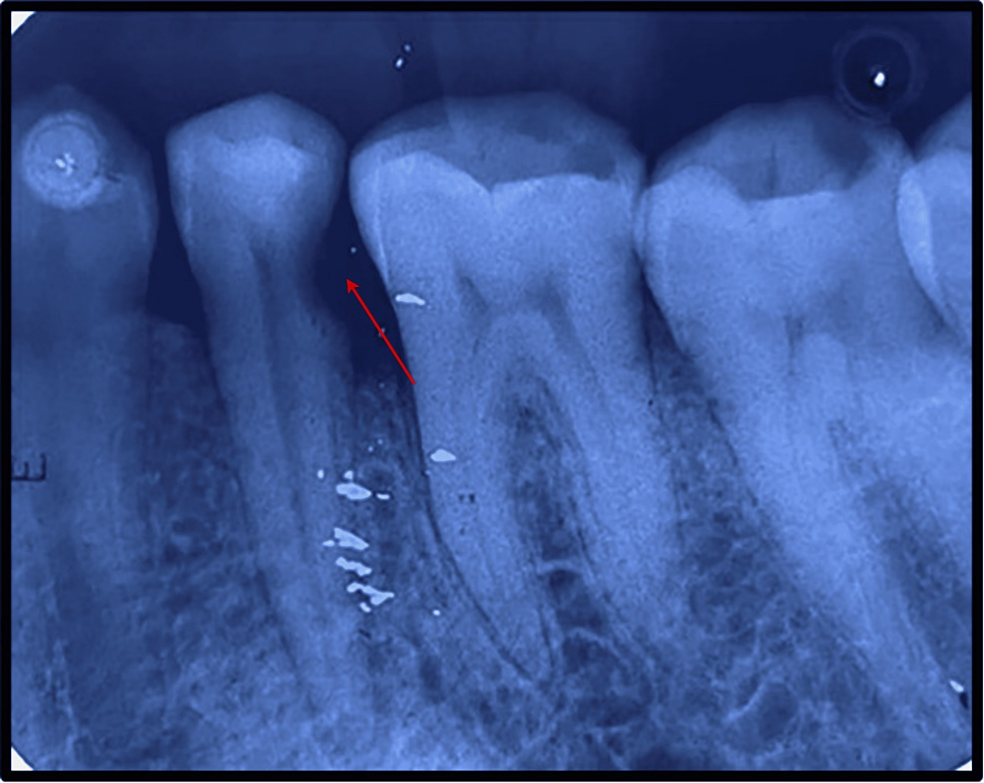
Pre-operative View Depicting Distant Proximal Caries With #35.

We present a case of a female labourer who reported to the Department of Endodontics at a rural hospital in Tertiary Care Central India in September 2020 with a chief complaint of pain in the lower left back region of the jaw over 7-10 days. Clinical examination revealed deep disto-proximal caries with respect to #35. The oral hygiene of the patient was acceptable, with no deleterious and parafunctional habits. The authors did not find any significant family or medical history that may affect the treatment course of the patient. Further, the pain was associated with the tooth on percussion, suggestive of periodontal inflammation, but the swelling or sinus tract was absent. To confirm the clinical finding, a pre-operative intra-oral periapical radiograph (IOPA) was taken, and it demonstrated minimal periapical changes along with slight widening of periodontal ligament (PDL) space and distoproximal caries involving pulp with #35 (Figure 1). The pulp vitality test showed no response. Hence, based on the findings and investigations (mentioned below), the diagnosis was confirmed as symptomatic irreversible pulpitis with apical periodontitis.

Investigations

- Intra-oral periapical radiograph in relation to #35

- Pulp vitality test using Electronic Pulp Tester (Parkell, Farmingdale, USA)

Treatment

**Figure 2:**
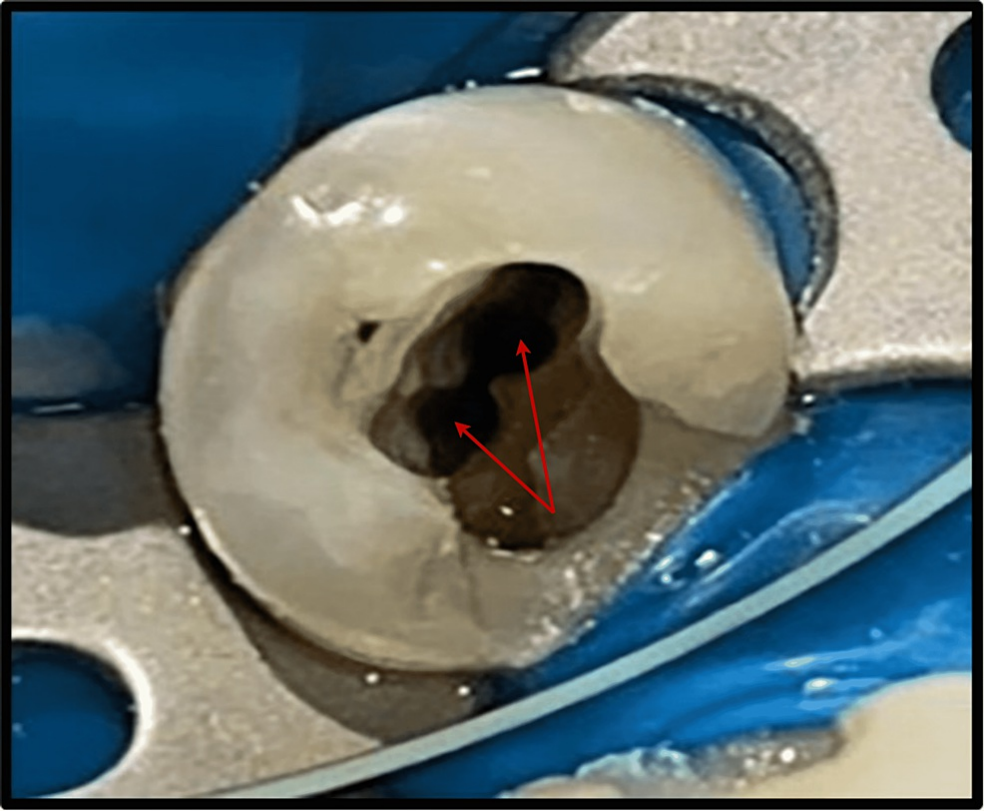
Access Cavity Preparation, Which Revealed Two Canal Orifices at the Pulpal Floor.

**Figure 3:**
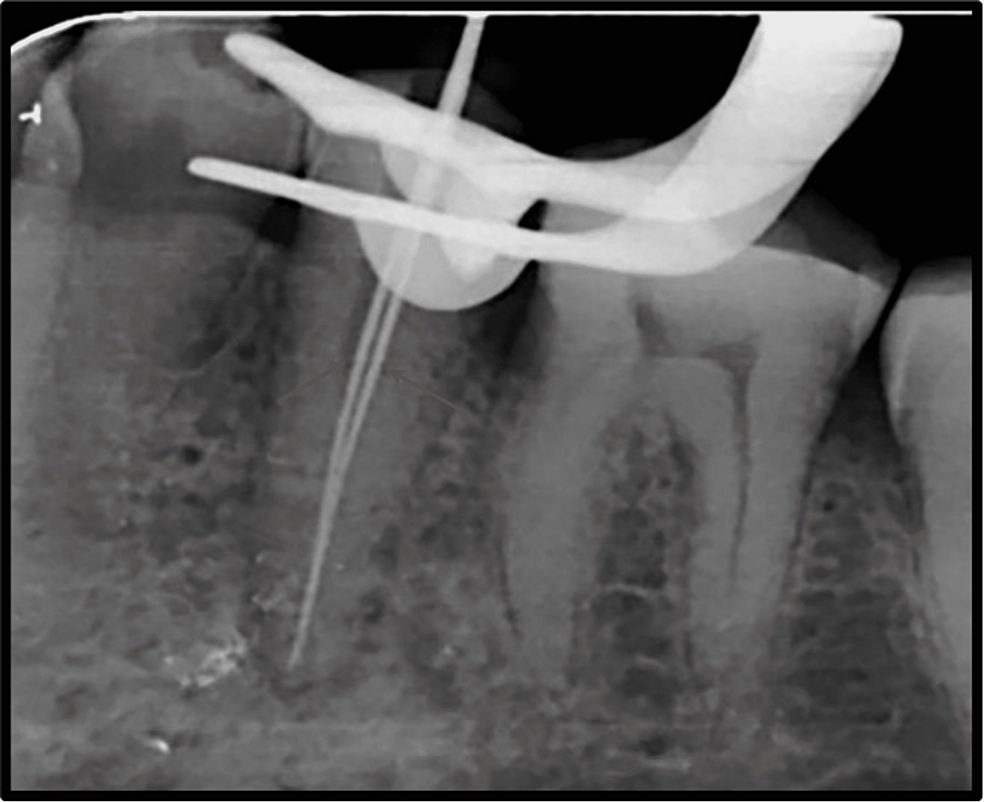
Working Length Determination.

**Figure 4:**
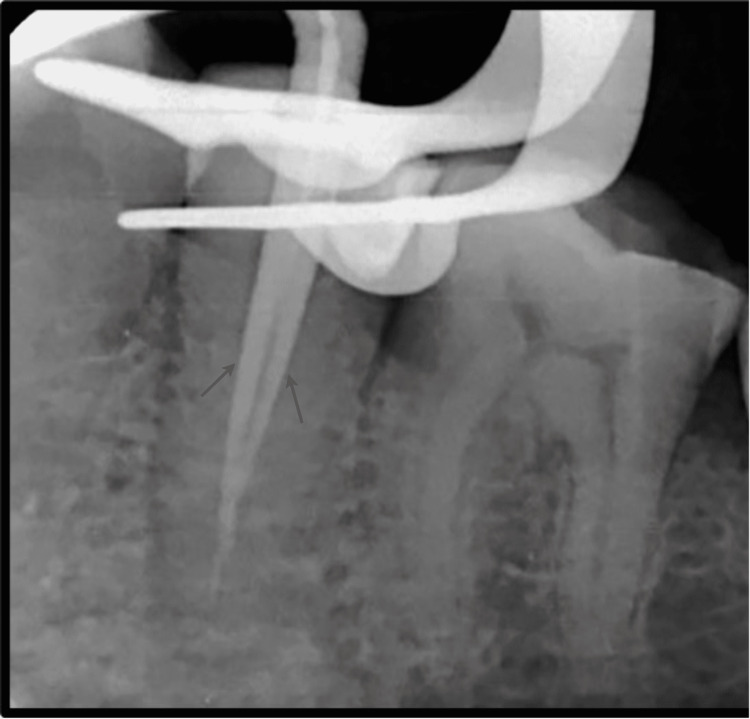
Evaluation of Master Cone Fit.

**Figure 5:**
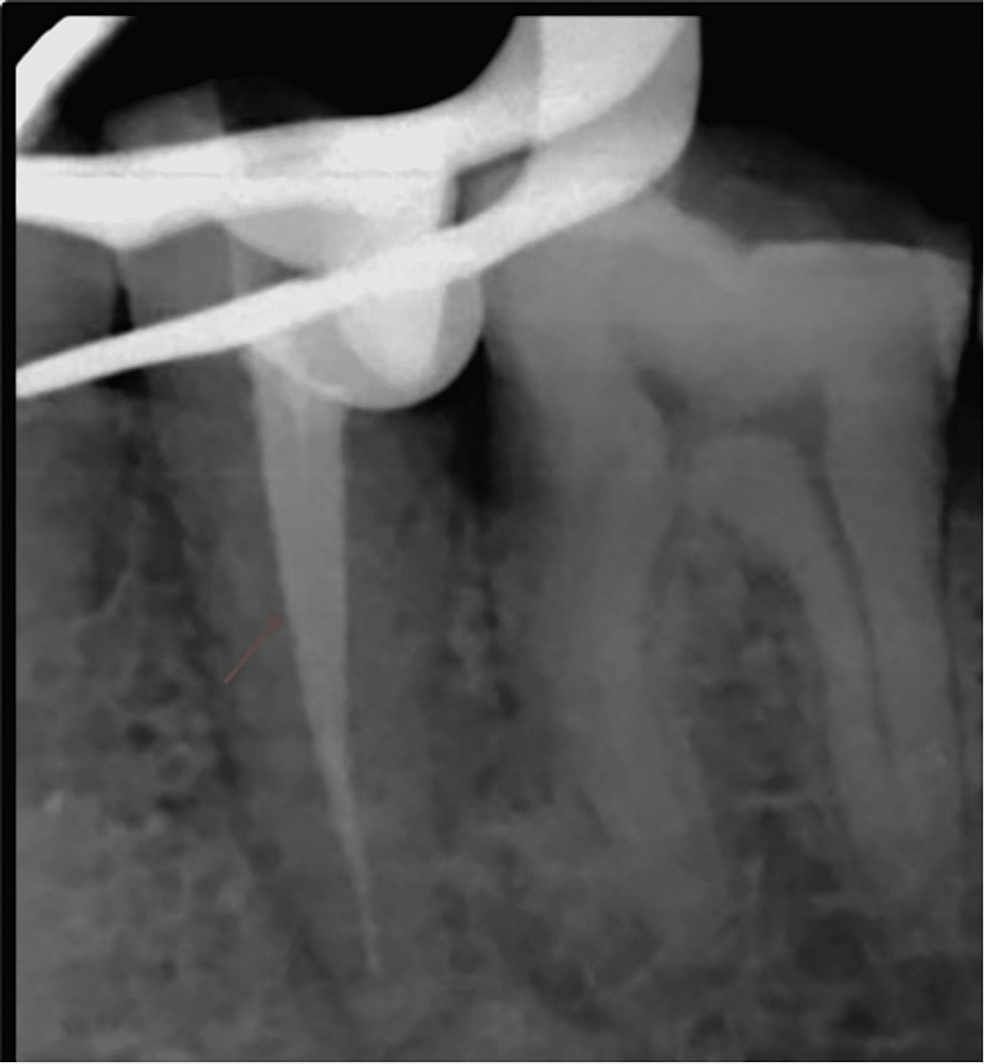
Obturation With an Inert Material to Achieve Hermetic Seal.

**Figure 6:**
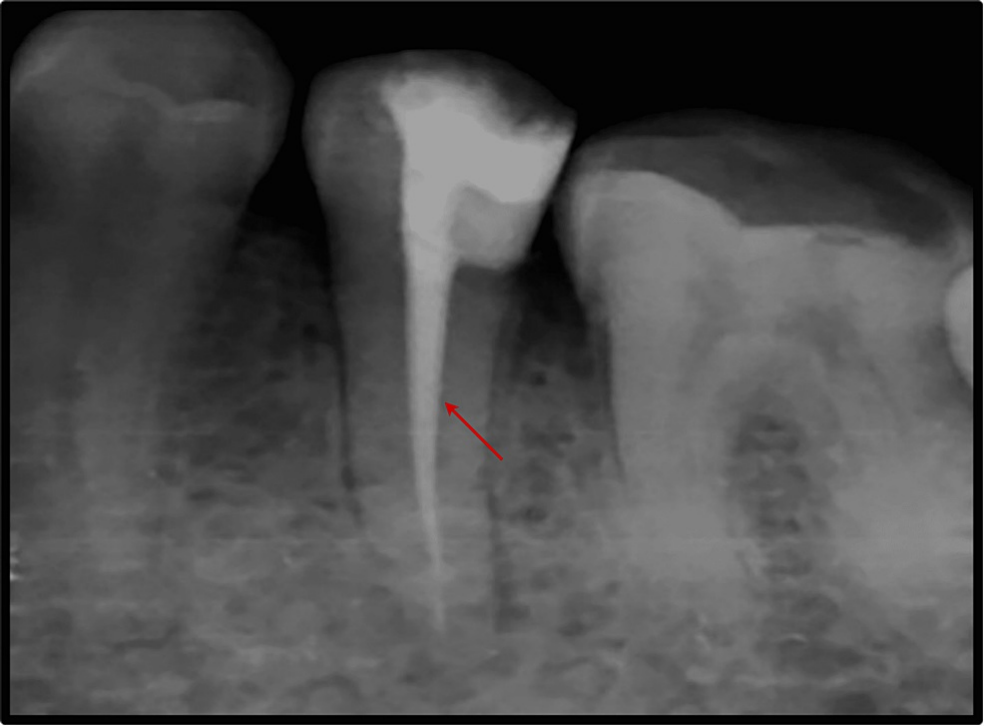
Immediate Post-Operative Follow-up Radiograph.

Root canal treatment was proposed for the patient, and informed consent was obtained before the initiation of treatment. Profound local anesthesia was achieved using lignocaine hydrochloride with adrenaline in the ratio 1:1,00,000. Under isolation, using a rubber dam (GDC Fine Crafted Dental Pvt. Ltd., India), complete distant proximal caries was removed, and access opening to expose the pulp canal orifice was done using 245, 169-L bur as shown in Figure 2. It was at this phase that the endodontist discovered an additional canal in #35, which is a very rare incidence. The patency of both the canals was confirmed with the #10 K file (Mani Inc., Tochigi, Japan). Subsequently, the initial working length was determined using the #15 K file (Mani Inc., Tochigi, Japan) with the help of Root Zx Mini Apex Locator (J. Morita Corp., USA), which was evaluated using a radiograph (Figure 3). Biomechanical preparation was done using hand-operated files and nickel-titanium rotary instruments (Dentsply Maillefer, Ballaigues, Switzerland). Thorough irrigation with 5.25% NaOCl, normal saline, and 0.2% chlorhexidine was also done simultaneously. The master cone fit was checked and confirmed on the radiograph following the final working length determination (Figure 4). Canals were dried using sterile paper points, and obturation was done with both the canals, followed by post-endodontic composite restoration (Figures 5-6). A postoperative radiograph was taken, and the complete hermetic seal was verified with a horizontally angulated post-treatment radiograph.

Outcome and follow up

As aforementioned, an immediate post-operative (IOPA) image was assessed, and it revealed that the two canals were completely filled. No abnormalities were apparent clinically and radiographically. The root canal therapy outcomes were achieved, and post-endodontic composite build-up was performed to fill the access cavity. Full coverage crown to #35 was delivered by the Department of Prosthodontics. The patient was followed-up at three months and six months. No obvious anomalies were noted, and the patient did not require any treatment interventions during the follow-up period. The patient was advised to maintain good oral hygiene.

## Discussion

Literature has documented a plethora of variations in canal configurations of the lower bicuspid. Due to such complex variations, root canal surgery of second premolars has a high failure rate. Even though these premolars have a configuration of 1-1, dental clinicians should always consider the probabilities of a more intricate canal pattern before initiating endodontic treatment procedures [5,6]. The configuration in the present case corresponds to the Vertucci type IV, which is two distinct canals extending from the pulp chamber to the apex [7]. The radiological findings show the presence of minor periapical alterations based on the bi-dimensional radiological images of the case presented. Many technological advancements have emerged to detect missed canals that cannot be apparent in IOPA. One such technique, the cone-beam computed tomography (CBCT), is helpful in the detection of any missed canal [8]. Three-dimensional vision provided by CBCT images allows the clinician to investigate anatomical features and disease alterations in detail. CBCT images have not been recorded in this case because of their unavailability during the procedure.

The use of magnifying loupes for inspection of the pulp chamber aids in the location of the root canals [9]. Furthermore, fiber optic illumination and sodium hypochlorite bubbling effects aid in the exploration of the intricate root canal system [10]. Altering the access cavity preparation using a Gates-Glidden (GG) drill increases the root canal accessibility and visibility, which is suggested in cases with atypical canal anatomy. Anatomical studies of three canals in the lower premolars have revealed two orifices on the buccal side and one on the lingual side [11]. Furthermore, in cases with unusual canal anatomy, such as in the present case, it is recommended that the biomechanical preparation of the apical third should be minimized to reduce the risk of iatrogenic preparation errors. 

According to the literature, the intracanal dressing should be given if a periapical lesion is present but in the present case, no intracanal dressing was given because we followed the protocol for single-visit endodontics. Because of the intricate anatomy along with limited access, using lateral compaction and standardized gutta-percha (GP) cones for obturation will not be considered in such cases [12]. Hence all the canals were filled by similar GP cones along with a vertical compaction method trailed by the final restoration for decreasing leakage [13]. If one of the canals is missed, it will serve as the nidus for microbial reinfection. This will lead to the failure of root canal treatment, and the infection may progress further in the periapical region, thereby causing conditions like periapical abscess, etc. Therefore to avoid such complications and tooth extraction, proper canal negotiation is very critical so that they can be discovered and treated successfully. Once the clinical and radiographical data is collected and analyzed, the level of difficulty is assessed, after which a decision is made to treat the case further or refer it to a specialist, if required.

## Conclusions

The current case illustrates the peculiar morphology of a mandibular bicuspid, serving as a reminder that these differences can occur during endodontic therapy and that the course of treatment will change accordingly. The mandibular second premolar varies in canal type and configuration, so the clinician must be aware of these variations. Finding the extra or additional canal requires performing a tactile examination. The additional and furcal canals, which serve as exit portals, are likewise sealed by three-dimensional obturation in addition to the apical third.
